# Randomized phase III trial comparing surgery alone to UFT + PSK for stage II rectal cancer (JFMC38 trial)

**DOI:** 10.1007/s00280-017-3466-7

**Published:** 2017-11-01

**Authors:** Kiyotaka Okuno, Toru Aoyama, Koji Oba, Noboru Yokoyama, Nobuhisa Matsuhashi, Katsuyuki Kunieda, Yoji Nishimura, Hiroki Akamatsu, Takaya Kobatake, Satoshi Morita, Takaki Yoshikawa, Junichi Sakamoto, Shigetoyo Saji

**Affiliations:** 1 Department of Surgery, Kindai University Faculty of Medicine, Osaka, Japan; 2 Department of Surgery, Yokohama City University Yokoham, 3-9 Fukuura, Kanazawa-ku, Yokohama, 236-0004 Japan; 3 Department of Biostatistics, The University of Tokyo, Tokyo, Japan; 4 Digestive Disease Center, Showa University Koto Toyosu Hospital, Tokyo, Japan; 5 Department of Surgical Oncology, Gifu University, Gifu, Japan; 6 Department of Surgery, Gifu Prefectural General Medical Center, Gifu, Japan; 7 Department of Gastroenterological Surgery, Saitama Cancer Cancer, Saitama, Japan; 8 Department of Surgery, Osaka Police Hospital, Osaka, Japan; 9 Department of Surgery, Shikoku Cancer Center Hospital, Matsuyama, Japan; 10 Department of Biomedical Statistics and Bioinformatics, Kyoto University Graduate School of Medicine, Kyoto, Japan; 11 Department of Gastrointestinal Surgery, Kanagawa Cancer Center, Yokohama, Japan; 12 Japanese Foundation for Multidisciplinary Treatment of Cancer, Tokyo, Japan; 13 Tokai Central Hospital, Kakamigahara, Japan

**Keywords:** Rectal cancer, PSK, UFT, RFS, OS

## Abstract

**Background:**

We conducted a randomized phase III trial comparing tegafur/uracil (UFT) and Polysaccharide-K (PSK) to surgery alone in curatively resected stage II rectal cancer patients.

**Methods:**

Patients were randomly assigned to receive either UFT and PSK or surgery alone in a 1:1 ratio with a minimization method to balance the treatment allocation. The primary end point of this study was the disease-free survival (DFS). The secondary end point was the overall survival (OS).

**Results:**

From October 2011 to February 2013, 111 patients were registered from 62 institutions. The study was prematurely closed due to poor accrual after reaching 20% of its goal. The patients’ characteristics were similar between the UFT and PSK group and the surgery-alone group. The DFS rate was 76.0% at 3 years and 65.1% at 5 years in the UFT and PSK arm and 84.0% at 3 years and 77.2% at 5 years in the surgery-alone arm. The DFS was slightly worse in the UFT + PSK arm than in the surgery-alone arm, but the difference did not reach statistical significance (log rank *p* = 0.102). The OS rate was 100% at 3 years and 97.9% at 5 years in the UFT + PSK arm, while that was 100% at 3 years and 93.4% at 5 years in the surgery-alone arm. The OS was similar in the UFT + PSK arm and surgery-alone arm (*p* = 0.533).

**Conclusion:**

The present study suggests that UFT and PSK are not attractive candidates to advance to the next phase III study because the DFS was slightly worse in the UFT and PSK arm than in the surgery-alone arm.

## Introduction

Colorectal cancer is the third-most commonly diagnosed cancer in males and the second-most commonly diagnosed cancer in females, with an estimated 1.4 million new cases and 693,900 deaths occurring in 2012 [1]. Approximately, one-third of these tumors arise in the rectum [2]. Complete resection is essential for achieving a cure of colorectal cancer. Epidemiological studies have reported that postoperative recurrence of rectal cancer is higher than that of colon cancer [3, 4]. For tumors confined to the rectal wall (including T1 and some T2 tumors), local excision can result in good local control while preserving the anal sphincter. However, most tumors are diagnosed at more advanced stages. The perioperative adjuvant treatment for stage III rectal cancer is internationally accepted as a standard treatment with established efficacy, but the usefulness of such treatment for stage II rectal cancer remains controversial [5, 6]. The major Western guidelines recommend adjuvant chemotherapy for “high-risk stage II” rectal cancer [7].

In Japan, tegafur/uracil (UFT; Taiho Pharmaceutical Co., Tokyo, Japan), an orally administered fluoropyrimidine inhibitor of dihydropyrimidine dehydrogenase that contains tegafur and uracil in a 1:4 molar ratio, recently showed a survival benefit in rectal cancer. Hamaguchi et al. conducted two independent randomized controlled trials in patients with stage III colon and rectal cancer (National Surgical Adjuvant Study of Colorectal Cancer, NSAS-CC trial). They found that postoperative adjuvant chemotherapy with UFT was tolerated and successfully improved the recurrence-free survival (RFS) and overall survival (OS) in patients with stage III rectal cancer. Furthermore, previous studies have shown favorable toxicity of adjuvant treatment with UFT. Therefore, the use of UFT as an adjuvant treatment was deemed an option for stage II rectal cancer [8].

Protein-bound polysaccharide K (KRESTIN, PSK; Kureha Chemical Industry Co., Tokyo, Japan), which is extracted from the mycelia of *Coriolus versicolor*, has immunomodulatory activities. It induces the production of interleukin (IL)-2 and interferon (IFN), thereby stimulating lymphokine-activated killer cells (LAK) and enhancing natural killer (NK) cells. A randomized, double-blind trial as well as a meta-analysis of those trials for resectable gastric cancers showed that PSK provided borderline significant benefits in improving the disease-free survival (DFS) and OS [9, 10]. The beneficial effects of PSK have been attributed to the activation of leucocyte chemotactic locomotion and phagocytic activity. With respect to colorectal cancers, the efficacy of adding PSK for standard cytotoxic chemotherapy has also been reported, mainly in colon cancers [10–13].

Given the above, to investigate the benefits of UFT plus immunochemotherapy with PSK, we conducted a randomized phase III trial comparing UFT and PSK to surgery alone in curatively resected stage II rectal cancer patients.

## Patients and methods

### Study design

The JFMC38 trial was a randomized, open-label, multicenter, phase III study. The trial was performed in patients diagnosed with stage II rectal cancer in Japan. Patients were randomly assigned to receive UFT and PSK or surgery alone in a 1:1 ratio with a minimization method to balance the treatment allocation according to the pathological stage (pT3 or pT4), primary tumor location (Ra or Rb), age (< 65 or ≥ 65 years of age), and medical center. The primary end point of this study was the DFS, and the secondary end point was the OS.

### Ethics

Study data and informed consent were obtained in accordance with the Declaration of Helsinki and approved by the Ethics Review Board of each institution. All patients were given a written explanation of the study, and they provided their written informed consent before participating.

### Inclusion and exclusion criteria

Tumors were staged according to the UICC version 6 [14]. The inclusion criteria were as follows: (1) Stage II, histologically confirmed adenocarcinoma of the rectum; (2) Patients who underwent curative surgery with ≥ D2 lymph node dissection; (3) Negative status for pathological lymph nodes; (4) Performance status of 0–2; (5) Not receiving pre-operative chemotherapy and/or radiation therapy; (6) Adequate hematologic, liver, and coagulation profiles (white blood cell [WBC] count ≥ 3,000/mm^3^ and ≤ 12,000/mm^3^, neutrophils count ≥ 1,500/mm^3^, platelet count ≥ 100,000/mm^3^, GOT ≤ 100 U/l and GPT ≤ 100 U/l, total bilirubin < 1.5 mg/dl, creatinine < 1.5 mg/dl, and normal ECG); (7) Able to start chemotherapy within 8 weeks after the operation; and (8) Provided consent to participate in this clinical study.

The exclusion criteria were as follows: (1) Unable to ingest anything or with digestive organ stricture; (2) Presence of serious coexisting morbidities; (3) Active synchronous or metachronous malignant disease; (4) Pregnant or lactating; (5) Not suitable for participating in the study for any other reason.

### Treatment methods

- Control arm: Surgery alone
- UFT-PSK arm: UFT was administered orally at 400 mg/m^2^/day thrice daily after meals for 5 days and followed by 2 days’ rest (one cycle) for 1 year after surgery. PSK was administered orally 3 g/day thrice daily after meals for 1 year after surgery.

### Follow-up

Patients were followed up in accordance with the protocol of the present study. Briefly, during protocol treatment, the clinical findings and laboratory data were evaluated every 2 weeks. After completion of the protocol treatment, patients were followed up in accordance with a predefined surveillance schedule until recurrence or death was confirmed for 5 years after surgery. Recurrence was assessed based on computed tomography (CT) scans. These tests were carried out every 4 months during the first 2 years after surgery and once every 6 months from the third year onward.

### Statistical analyses

The DFS at 5 years was predicted to be 75% in the surgery-alone arm and 85% in the PSK + UFT arm with assumption of a dropout rate of 5%, recruitment period for 36 months and additional follow-up period for 60 months. We calculated that a total enrollment of 540 patients was needed using a log-rank test, a two-sided alpha of 5%, and a statistical power of 80%.

The background characteristics of the postoperative clinical and pathological parameters between the UFT + PSK arm and surgery-alone arm are shown as percentages for categorical variables and the median with the range for continuous variables. The OS was defined as the period between randomization and any cause of death. The DFS was defined as the period between randomization and the occurrence of recurrence, second cancer, or death, whichever came first. The data for patients who had not experienced an event were censored at the date of the final observation. The OS and DFS curves were calculated using the Kaplan–Meier method, and were compared by the stratified log-rank test (stratification factors: pathological T stage, primary tumor location, and age). A Cox proportional hazards model was used to estimate the adjusted hazard ratio, with adjustment for the same stratification factors from the log-rank test as covariates. In addition, a multivariate Cox proportional hazards model was created using the stratification factors and other factors (sex, tumor diameter, lymph node metastasis, lymphadenectomy, lymphatic invasion, and vascular invasive) as covariates. Other factors were selected based on a priori knowledge. Subgroup analyses based on the stratification factors (pathological T stage, primary tumor location, and age) and other factors (sex, tumor diameter, lymph node metastasis, lymphatic invasion, and vascular invasive) were conducted, and the interaction between the treatment effect and each stratification factor was evaluated for the DFS. A two-sided *p* < 0.05 was defined as indicating statistical significance. The SAS software program, version 9.4 (SAS Institute Inc., Cary, NC, USA), was used for all statistical analyses. This study was approved by the ethics committee of the Japanese Foundation for Multidisciplinary Treatment of Cancer.

## Results

### Recruitment and patients’ characteristics

From October 2011 to February 2013, 200 institutions collaborated with the JFMC-38 study, and 111 patients were registered from 62 institutions. The study was prematurely closed due to poor accrual after reaching 20% of its goal. At the time of the analysis in June 2017, 7 patients had died, and 29 had experienced the primary event. Due to this low number of observed primary events, the power for the formal statistical analysis was limited.

**Fig. 1:**
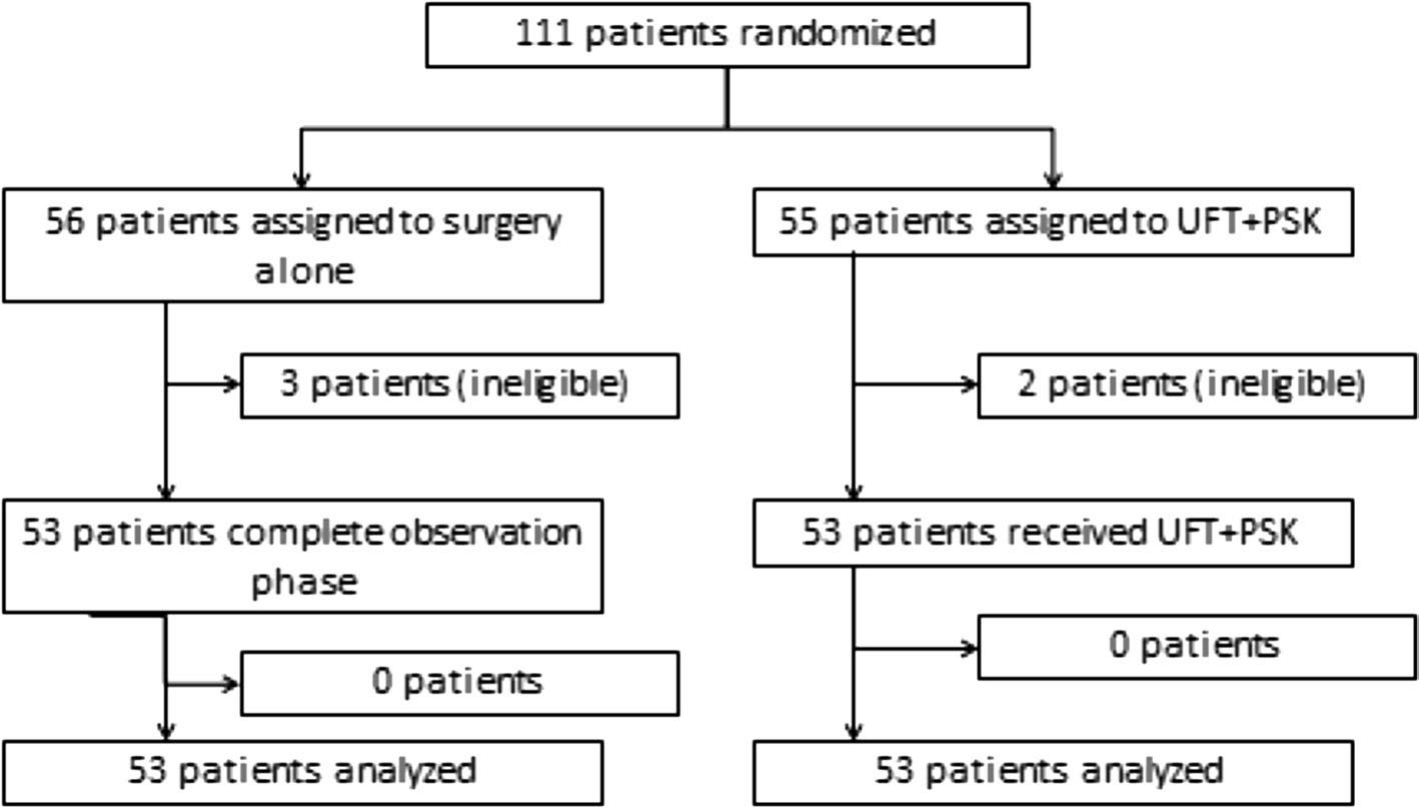
Consort diagram of the present study

Among 111 patients, 55 were allocated to the UFT + PSK arm and 56 to the surgery-alone arm. Figure 1 shows the consort diagram of the present study. After randomization, 5 patients (2 in the UFT + PSK arm and 3 in the control arm) were found to be ineligible. Primary analyses were based on data from all randomly assigned patients, excluding those who were ineligible. The two groups were well balanced with regard to the baseline clinical characteristics and pathological findings (Table 1). The median duration of follow-up was 5.3 years.

**Table 1:** Patient characteristics

Characteristics	Total (*n* = 106)	Surgery-alone group (*n* = 53)	UFT + PSK group (*n* = 53)
	No. of patients (%)	No. of patients (%)	No. of patients (%)
Age			
< 65 years	51 (48.1)	25 (47.2)	26 (49.1)
> 65 years	55 (51.9)	28 (52.8)	27 (50.9)
ECOG performance status			
0	99 (93.4)	47 (88.7)	52 (98.1)
1–2	7 (6.6)	6 (11.3)	1 (1.9)
Tumor location			
Ra	56 (52.8)	29 (54.7)	27 (50.9)
Rb	49 (46.3)	23 (43.4)	26 (49.1)
Missing	1 (0.9)	1 (1.9)	0 (0)
Tumor diameter (mm)			
Median	51.5	52.5	50
Range	19–140	25–110	19–140
Pathological T factor			
T3	95 (89.6)	45 (84.9)	50 (94.3)
T4	10 (9.5)	7 (13.2)	3 (5.7)
Missing	1 (0.9)	1 (1.9)	0 (0)
Lymphatic invasion			
Negative	56 (52.8)	26 (49.1)	30 (56.6)
Positive	49 (46.3)	26 (49.1)	23 (43.4)
Missing	1 (0.9)	1 (1.9)	0 (0)
Vascular invasion			
Negative	39 (36.8)	15 (28.3)	24 (45.3)
Positive	66 (62.3)	37 (69.8)	29 (54.7)
Missing	1 (0.9)	1 (1.9)	0 (0)
Histological type			
Differentiated	102 (96.4)	51 (96.2)	51 (96.2)
Undifferentiated	4 (3.6)	2 (3.8)	2 (3.8)

*ECOG* Eastern Cooperative Oncology Group

### Safety and feasibility

Table 2 show the adverse events reported in the present study. The most commonly reported adverse events of any grade in the UFT + PSK arm were elevated serum bilirubin nausea, vomiting, and fatigue. The adverse events of grade 3 or 4 that were more frequent in the UFT + PSK arm were elevated aspartate aminotransferase and alanine aminotransferase.

**Table 2:** Relevant adverse events

Adverse event	Surgery alone group					UFT + PSK group				
	Grade1	Grade2	Grade3	Grade4	Grade 3 or 4	Grade1	Grade2	Grade3	Grade4	Grade 3or 4
	Number of patients				(%)	Number of patients				(%)
Hematological										
Leukopenia	1 (1.9)	0 (0)	0 (0)	0 (0)	0 (0)	5 (9.4)	2 (3.8)	0 (0)	0 (0)	0 (0)
Neutropenia	0 (0)	0 (0)	0 (0)	0 (0)	0 (0)	0 (0)	7 (13.2)	0 (0)	0 (0)	0 (0)
Anemia	9 (17.0)	2 (3.8)	0 (0)	0 (0)	0 (0)	7 (13.2)	3 (5.7)	1 (1.9)	0 (0)	1 (1.9)
Thrombocytopenia	3 (5.7)	0 (0)	0 (0)	0 (0)	0 (0)	4 (7.5)	1 (1.9)	0 (0)	0 (0)	0 (0)
No hematological										
Elevated ALT	8 (15.1)	0 (0)	0 (0)	0 (0)	0 (0)	8 (15.1)	2 (3.8)	2 (3.8)	0 (0)	2 (3.8)
Elevated AST	5 (9.4)	0 (0)	1 (1.9)	0 (0)	1 (1.9)	7 (13.2)	1 (1.9)	2 (3.8)	0 (0)	2 (3.8)
Elevated serum Bil	2 (3.8)	1 (1.9)	0 (0)	0 (0)	0 (0)	10 (18.9)	6 (11.3)	0 (0)	0 (0)	0 (0)
Elevated creatine	3 (5.7)	0 (0)	0 (0)	0 (0)	0 (0)	1 (1.9)	3 (5.7)	0 (0)	0 (0)	0 (0)
Stomatitis	0 (0)	0 (0)	0 (0)	0 (0)	0 (0)	1 (1.9)	2 (3.8)	0 (0)	0 (0)	0 (0)
Nausea/vomiting	0 (0)	0 (0)	0 (0)	0 (0)	0 (0)	9 (17.0)	2 (3.8)	1 (1.9)	0 (0)	1 (1.9)
Diarrhea	1 (1.9)	0 (0)	0 (0)	0 (0)	0 (0)	9 (17.0)	2 (3.8)	1 (1.9)	0 (0)	1 (1.9)
Rash	0 (0)	0 (0)	0 (0)	0 (0)	0 (0)	3 (5.7)	3 (5.7)	0 (0)	0 (0)	0 (0)
Pigmentation	0 (0)	0 (0)	0 (0)	0 (0)	0 (0)	3 (5.7)	1 (1.9)	0 (0)	0 (0)	0 (0)
Anorexia	1 (1.9)	0 (0)	0 (0)	0 (0)	0 (0)	8 (15.1)	2 (3.8)	0 (0)	0 (0)	0 (0)
Fatigue	1 (1.9)	0 (0)	0 (0)	0 (0)	0 (0)	8 (15.1)	0 (0)	0 (0)	0 (0)	0 (0)

Among the 53 patients in the safety population who received UFT + PSK, the relative dose intensity (RDI) of UFT was > 90% in 37 patients (69.8%), 75–90% in 6 patients (11.3%), 50–75% in 5 patients (9.4%), and < 50% in 3 patients (5.7%), and data were missing in 2 patients (3.8%). The RDI of PSK was > 90% in 39 patients (73.6%), 75–90% in 5 patients (9.4%), 50–75% in 4 patients (7.6%), and < 50% in 3 patients (5.7%), and data were missing in 2 patients (3.8%). The reasons for withdrawal of treatment in the UFT + PSK arm included refusal of the patient to continue treatment because of adverse events or other factors in nine patients, the detection of metastasis or relapse in six, and other reasons in six.

### Survival analyses

**Fig. 2:**
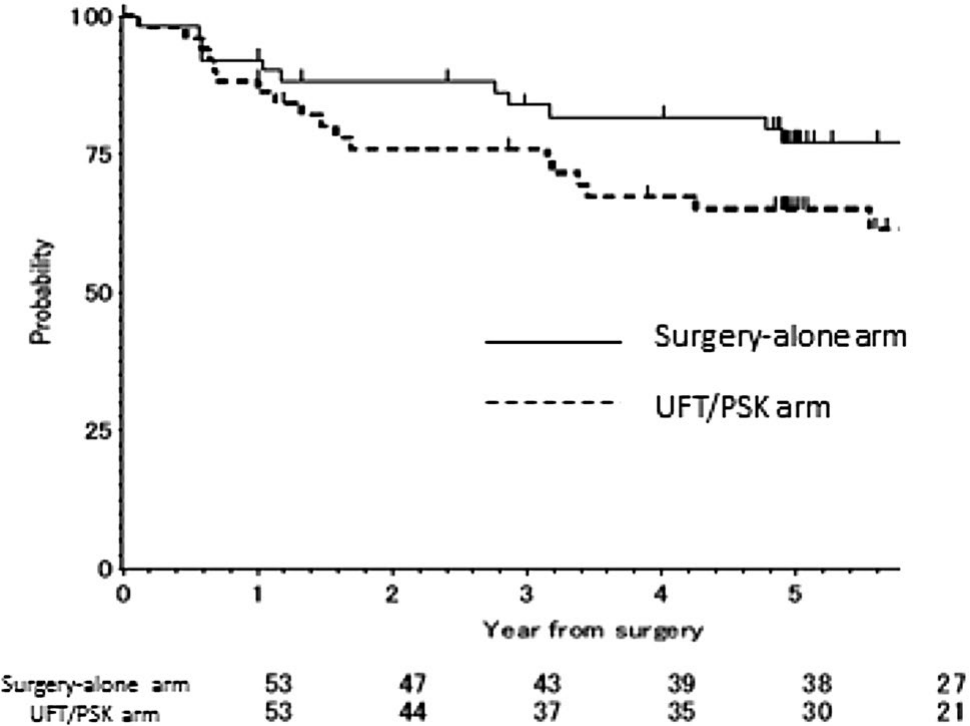
A comparison of the recurrence-free survival between the UFT/PSK arm and surgery-alone arm

The DFS rate was 76.0% at 3 years and 65.1% at 5 years in the UFT + PSK arm and 84.0% at 3 years and 77.2% at 5 years in the surgery-alone arm. The DFS was slightly worse in the UFT + PSK arm than in the surgery-alone arm, but the difference did not reach statistical difference (log rank *p* = 0.102). The DFS curves are shown in Fig. 2. The hazard ratio for DFS was 1.94 (95% confidence interval [CI], 0.90 to 4.18; *p* value = 0.092). In the multivariate Cox proportional hazards model, the adjusted hazard ratio for DFS was 2.45 (95% CI, 0.99 to 6.03; *p* value = 0.052). In the subgroup analysis, UFT + PSK was statistically inferior to the control arm (hazard ratio = 2.85; 95% CI, 1.01 to 8.03; *p* value = 0.048), but there were no statistically significant interactions between the treatment effect and either subgroup. Table 3 showed the site of first relapse according to treatment group.

**Table 3:** Site of first relapse, according to treatment group

Total number of relapse	Total (*n* = 29)	Surgery alone group (*n* = 11)	UFT + PSK group (*n* = 18)
	(%)	(%)	(%)
Local	8 (27.6)	3 (27.2)	5 (27.8)
Lymph node	2 (6.9)	1 (9.1)	1 (5.6)
Hematogenous			
Lung	5 (17.2)	1 (9.1)	4 (22.2)
Liver	4 (13.8)	1 (9.1)	3 (16.7)
Others	10 (34.5)	5 (45.5)	5 (27.8)

**Fig. 3:**
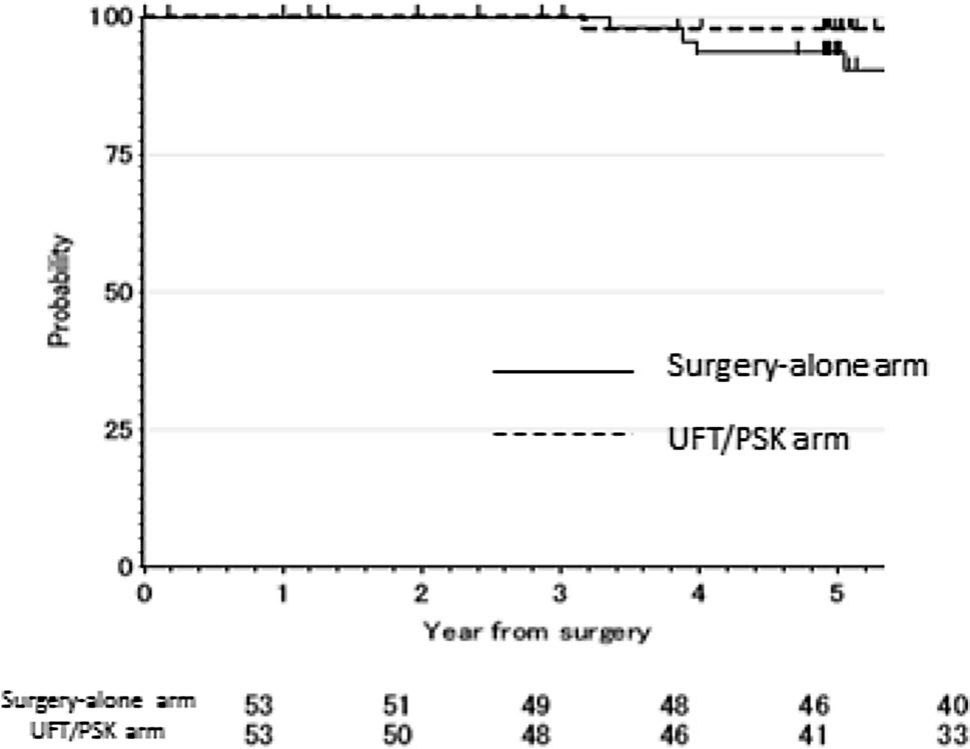
A comparison of the overall survival between the UFT/PSK arm and surgery-alone arm

The OS rate was 100% at 3 years and 97.9% at 5 years in the UFT + PSK arm and 100% at 3 years and 93.4% at 5 years in the surgery-alone arm. The OS was similar in the UFT + PSK arm and surgery-alone arm (*p* = 0.533). The OS curves are shown in Fig. 3. The hazard ratio for the OS was 1.82 (95% CI, 0.38 to 8.74; *p* value = 0.454).

## Discussion

The present randomized phase III study aimed to compare the outcomes of UFT and PSK to those of surgery alone in curatively resected stage II rectal cancer patients. The primary purpose of this study was to confirm the efficacy of UFT and PSK in improving the DFS. The results showed that UFT and PSK did not improve the DFS compared with surgery alone in 20% of the planned sample size. Thus, confirmative results could not be obtained from this study. However, the present findings do suggest that UFT and PSK were not attractive candidates for advancing to a test arm in the next phase III study, as the hazard ratio for the DFS was 1.94 against surgery alone.

First, we want to discuss why the patient accrual was so poor. Some patients avoided registering for the present clinical trial due to the adverse events of UFT. UFT induces both hematological and non-hematological toxicities, such as anorexia, fatigue, and nausea and affects the quality of life of patients. Therefore, even though the physicians explained the details and importance of the clinical trial to the patients, many avoided registering. In addition, some physicians avoided registering patients for the present clinical trial because they wanted to prescribe another adjuvant chemotherapy regimen. The major Western guidelines recommend adjuvant chemotherapy, such as oxaliplatin-based chemotherapy, for “high-risk stage II” rectal cancer [7]. Therefore, even though patients were members of the target population, physicians might have avoided encouraging their registration in the present clinical trial for “high-risk stage II” rectal cancer. As the study was terminated due to poor accrual, the results failed to show the survival superiority of the test arm.

Second, we want to discuss why the DFS of PSK and UFT was not sufficient to support the application of this regimen as an adjuvant treatment for stage II rectal cancer. Given the results of previous studies such as NSAS-CC, we selected UFT as the adjuvant chemotherapy agent for the present study [8]. Non-specific immunomodulators have been considered particularly promising anticancer agents when combined with chemotherapy or chemoradiotherapy for curatively resected gastrointestinal cancers in an adjuvant setting [9, 12], and PSK, OK-432, and Lentinan are now widely used in Japan. With regard to locally advanced gastric cancers, a number of immunochemotherapy clinical trials combined mainly with oral fluorinated pyrimidine anticancer agents have been conducted. A borderline significant effect was demonstrated in randomized clinical trials as well as in a meta-analysis for PSK and OK-432 [9, 10, 15, 16, 15, 16], although no significant effect of Lentinan was proven for advanced or metastatic gastric cancers in a recently published study [17]. Although PSK has shown somewhat favorable outcomes for colon cancer patients in adjuvant settings, initial studies for those agents failed to confirm their significant advantage, and they were eventually excluded from use in community practice in early development. However, the primary end point was not met in the present study.

Some discrepancies were observed in the findings for the DFS and OS in our study. Although the difference did not reach significance, the DFS was slightly worse in the UFT + PSK arm than in the surgery-alone arm. In contrast, the OS rates were similar in the UFT + PSK arm and surgery-alone arm. This discrepancy might be due to treatments after recurrence, especially chemotherapy, prolonging the survival of these patients. Indeed, chemotherapy for recurrence certainly prolongs the OS [18, 19].

In conclusion, the findings from the present study suggest that UFT + PSK was not attractive candidates to advance to the next phase III study because the DFS was slightly worse in the UFT and PSK arm than in the surgery-alone arm.
